# Frontloading selectivity: A third way in scientific publishing?

**DOI:** 10.1371/journal.pbio.3000693

**Published:** 2020-03-25

**Authors:** Christopher D. Chambers

**Affiliations:** Cardiff University Brain Research Imaging Centre (CUBRIC), School of Psychology, Cardiff University, Cardiff, Wales, United Kingdom

## Abstract

Prestigious scientific journals traditionally decide which articles to accept at least partially based on the results of research. This backloaded selectivity enforces publication bias and encourages authors to selectively report their most persuasive findings, even when they are misleading, biased, and unreliable. One answer to backloaded selectivity is to curtail editorial selectivity altogether, deciding publication on the basis of technical merit alone. However, this strategy is unlikely to win appeal among highly selective journals. A third way is to frontload selectivity—reaching editorial decisions based on rigorous evaluation of the research question and methodology but before the research is conducted and thus regardless of the eventual results. This model, now offered at *PLOS Biology* in the form of “Preregistered Research Articles” (or Registered Reports), allows a scientific journal to maintain high selectivity for the importance and rigor of research while simultaneously eliminating outcome bias by editors, reviewers, and authors. I believe the rise of Registered Reports among selective journals will change how research is evaluated and may trigger the realization that frontloaded selectivity is the most secure way of advancing knowledge.

## Abstract 2

This Perspective article introduces the concept of "frontloaded" selectivity as a means for journals to remain highly discerning while also eliminating publication bias. The newly launched "Preregistered Research Article" format at PLOS Biology (AKA Registered Reports) is a working example of this principle.

What does it mean for a scientific journal to be highly selective? To anyone outside science, the notion of “selectivity” conjures images of a competition to choose the “best” of something—in this case, the research of the very highest quality. But is that what a highly selective journal actually does? What if selectivity means something else?

To illustrate, consider the following thought experiment. Two research teams in your field independently perform the same set of experiments to test an important hypothesis. Each team uses the same cutting-edge, rigorous methodology. After conducting their experiments to the same (exemplary) standard, one team confirms the hypothesis with striking clarity, reporting beautiful, unequivocal results. The other team finds mostly null, complicated, and inconsistent results that do not yield a strong conclusion. Each group prepares a separate manuscript based on their findings. Now ask yourself: would both manuscripts have the exact same chance of being published in your field’s most selective, prestigious journals?

If the answer is yes, then congratulations—your field is doing well. Stop reading now and get back to doing science. If, more likely, the answer is no—and not just no but “not in a million years would these manuscripts have the same chance of being published; in fact, the one with null results might never even be submitted because experienced scientists in my field know better than to even try publishing such results”—then keep reading, and congratulations, your field belongs to a very broad, very inclusive club: fields suffering from publication bias.

Publication bias is a form of outcome bias that occurs when, all else being equal, gatekeepers decide what to publish (or what to submit for publication) based on the results. It arises because in many sciences, the contribution that a study makes to a field—its impact or newsworthiness—is often judged not only on what the researchers did but also on what they found.

A quick scan of the policies of the most prominent journals in science reveals this value system in full glory. Selected papers will be the most “influential,” the most “original,” the “most important” (or of “exceptional importance”) to specialist and nonspecialist researchers; they will be work that has the “greatest potential impact” both within and between disciplines, the most “novel” and the most “significant.” The competition for publishing in such journals is extreme, with only 5%–10% of submissions making the cut.

Notice what is not mentioned. Reproducibility. Transparency. Rigor. Quality. Of course, this does not mean that these characteristics are irrelevant. Scientists know they will need to reach some essential minimum in these areas to have a look-in. But their absence signals to authors that these dimensions are not enough to distinguish the winners from the losers. Of the manuscripts that meet whatever quality standard applies, those that succeed in the competition for “space” will be the ones that also report the most beautiful, novel, clear, striking, groundbreaking, unexpected, remarkable, thought-provoking, persuasive, game-changing results.

Selecting what science should appear with what prominence in the scientific record based on results is what we can call backloaded selectivity. We have known for decades that this kind of bias distorts the evidence base by deprioritizing negative or null results arising from studies of equal quality, and—possibly worse—incentivizes researchers to leave no stone unturned in forcing their results to tell a good story. Backloaded selectivity transforms the research environment into a breeding ground for *p*-hacking, hindsight bias, and other forms of selective reporting that litter journals with irreproducible findings [1].

Why, given the terrible consequences of backloaded selectivity, do prestigious journals persist in it? I believe there are 2 main reasons, one deliberate and one accidental. The first is that, deep in their (often corporate) identities, many prestigious journals see themselves less as records of science and more as news outlets drawing attention to spectacular observations. Results are currency, currency is power, and the most prominent journals strive to channel that power. This indulgence—reinforced by academia’s fetishization of such journals—then allows the editorial leadership to rationalize publication bias as a community service. “We don’t promise that what we publish is true—what journal can—but we guarantee it will turn heads.” The regretful editor, desperate to reject 9 out of 10 submissions, tells the unlucky (or especially scrupulous) author that while their submission is “methodologically sound,” the “findings do not represent a sufficiently major advance” to warrant publication in the journal, punctuated with the solemn recommendation that the author should consider submitting their solid, albeit boring results to a “more specialized” journal. Specialist journals, not wanting to miss out on the big leagues, then start applying the same formula, and before you know it, any null result is so specialized as to out-specialize even the most specialist journal.

The second reason why backloaded selectivity persists is invisible cognitive bias. When expert reviewers see null results, they are more likely to go on the hunt for imperfections in the methodology or rationale [2]. This bias is especially insidious because although it is thoroughly results-driven, it requires no explicit reference to the results at all. With reviews in hand that focus attention solely on a litany of methodological problems, the editor can comfortably reject the manuscript due to “fatal flaws,” and if they should ever be challenged for this decision, they can claim (quite correctly) that the results had no explicit role in the journal’s decision. Of course, the editor is either ignoring or ignorant of the possibility that it was the reviewer’s knowledge of the results—and consequent motivated reasoning—that led to the application of a double standard in the assessment of methodology. The specter of outcome bias lurks in the corner, silent and deadly.

For some journals, the solution to backloaded selectivity is to reduce selectivity altogether, deciding which manuscripts are published purely on the basis of “technical merit.” As praiseworthy as this is, such journals then predictably become seen by many (particularly senior) scientists as dumping grounds for lower-quality science. “The presence of a *PLOS ONE* or *Frontiers* paper in your track record only dilutes your résumé,” intones the professor (irrationally) to their trainee, but the advice can stick. Reducing selectivity overall no doubt helps reduce publication bias, but it also fuels the impression that the journal (and the researcher) will publish anything that passes some minimal quality threshold. To many scientists, this is a feature of good practice, because there are already numerous prestudy filters for selecting the research that is worth attempting. But to the older generation especially, it can be regarded as a bug, and to prestigious journals, it is alien.

The question, then, is whether there could be a unifying third way that enables a prestigious journal to maintain the same degree of selectivity while eradicating publication bias. Registered Reports (RRs), now offered by *PLOS Biology* under the name “Preregistered Research Articles” [3], provide the answer, not by reducing selectivity but by replacing backloaded selectivity with frontloaded selectivity. For an RR, the most significant and substantial part of the review process occurs before the research is undertaken during so-called Stage 1 review. Studies with a compelling rationale and rigorous methodology are then provisionally accepted for publication regardless of the eventual results, provided the authors adhere closely to their approved protocol [4].

The RR model is important because it enables journals to embrace selectivity in a way that protects, rather than threatens, their sense of reputation and prestige. Instead of conditioning even implicitly on unstable findings, it prompts the reviewers and editors to probe more fundamental issues. Which questions are the most important for our field to answer? What methods of answering these questions are the most rigorous and insightful? Which studies are so well designed and so theoretically incisive that any obtained result will significantly advance knowledge? The assumption behind RRs is that rather than focusing on the outcomes, it is by scrutinizing the process blind to those outcomes that we direct laser-like precision on one thing that matters most in scientific evaluation: quality.

From my perspective, as one of the cofounders of RRs, I find it fascinating how the most prestigious journals have responded to the presence of RRs in the publishing landscape. When we first launched the initiative in 2013, many senior scientists dismissed out of hand the possibility of the format being offered by even one major journal. The received wisdom was that because such journals exert an iron grip over which results they will publish, RRs would always be an impossible proposition. But the critics were wrong, and instead, we are seeing the selection process at Stage 1 being deployed in interesting ways to invest in the predicted value of future results.

For example, *PLOS Biology*, like the founding journal *Cortex*, explicitly judges Stage 1 manuscripts according to “the importance of the research question,” on the assumption that studies asking more important questions will generate more important answers. Journals such as *Nature Human Behaviour* go one step further, assessing “the importance of the research question(s) and relevance for a broad, multidisciplinary audience.” Other outlets, such as *PLOS ONE*, *Royal Society Open Science (RSOS)*, and *F1000Research*, eschew judgments of importance altogether, replacing this criterion with “the scientific validity of the research question” (*RSOS*) or by instead asking reviewers “is the rationale for, and objectives of, the study clearly described?” (*F1000Research*) In this way, a Stage 1 RR that *PLOS Biology* or *Nature Human Behaviour* might consider too incremental or specialized—for example, replicating a well-known phenomenon in order to establish an unbiased parameter estimate—would, if methodologically valid and based on a clear rationale, be routinely accepted and published in *RSOS*, *F1000Research*, or *PLOS ONE*.

Another way selectivity can be frontloaded at Stage 1 is through the strength of statistical evidence required. *Nature Human Behaviour* requires all prespecified hypothesis tests to achieve 95% power to detect the smallest effect size of interest or to feasibly achieve Bayes factors (BF) >10 (strong evidence). At *Cortex*, the threshold is power of 90% and BF > 6 (moderate evidence), whereas *PLOS Biology* sits in the middle, requiring power of 90% and BF > 10. *RSOS*, on the other hand, sets no threshold at all, on the grounds that research lacking statistical power or evidential strength can still make an important contribution to knowledge, especially when deploying expensive methods or in hard-to-reach populations. One small but unbiased brick in the wall is better than a house made of paper.

With RRs now offered by over 200 journals, the goal of pushing the format into the mainstream has been achieved. This means we can now observe how prominent journals such as *PLOS Biology* go about frontloading selection. Given only the theory and methods, how will reviewers and editors decide which science is worthy of in-principle acceptance? Which questions will be judged the most important and why? What new means and standards will emerge for assessing research quality? Which prominent journals will follow? *PLOS Medicine*? *Science*? *PNAS*? *Nature*? That they will join this evolution in scientific evaluation now seems inevitable; the only question is when and on what terms, and what happens next.

And what happens next may be an even deeper realization. In law, it is said that justice must be blind to wealth and power, or it is no longer just. Insofar as the results of research are a currency, then the normalization of RRs may lead us to conclude the same truth about science: that we must judge research blind to results, or we are no longer producing knowledge.

## Abbreviations

BF: Bayes Factor
RR: Registered Report
RSOS: Royal Society Open Science
